# Adaptive Fault-Tolerant Tracking Control for Multi-Joint Robot Manipulators via Neural Network-Based Synchronization

**DOI:** 10.3390/s24216837

**Published:** 2024-10-24

**Authors:** Quang Dan Le, Erfu Yang

**Affiliations:** Robotics and Autonomous Systems Group, Department of Design, Manufacturing and Engineering Management, University of Strathclyde, Glasgow G1 1XJ, UK; l.quang-dan@strath.ac.uk

**Keywords:** fault-tolerant control, passive fault-tolerant control, robot manipulator, adaptive control, synchronization, neural network

## Abstract

In this paper, adaptive fault-tolerant control for multi-joint robot manipulators is proposed through the combination of synchronous techniques and neural networks. By using a synchronization technique, the position error at each joint simultaneously approaches zero during convergence due to the constraints imposed by the synchronization controller. This aspect is particularly important in fault-tolerant control, as it enables the robot to rapidly and effectively reduce the impact of faults, ensuring the performance of the robot when faults occur. Additionally, the neural network technique is used to compensate for uncertainty, disturbances, and faults in the system via online updating. Firstly, novel robust synchronous control for a robot manipulator based on terminal sliding mode control is presented. Subsequently, a combination of the novel synchronous control and neural network is proposed to enhance the fault tolerance of the robot manipulator. Finally, simulation results for a 3-DOF robot manipulator are presented to demonstrate the effectiveness of the proposed controller in comparison to traditional control techniques.

## 1. Introduction

During the past few decades, robots have played and continue to play an important role in many areas, especially in the advancement of various industries and replacement of humans in harsh and hazardous work environments. Consequently, they have been increasingly studied and developed to enhance their working capabilities and safety in many circumstances and diverse work environments. In robot manipulator systems, the occurrence of faults in actuators or sensors can reduce the performance of the robot, damage the system, and have economic implications [1,2]. Therefore, fault-tolerant control (FTC) is introduced to improve the safety of robot systems. It guarantees the acceptable performance and provides operators with a scheduled maintenance time to ensure smooth operation of the robotics system. In general, fault-tolerant control can be divided into two types: (1) active fault-tolerant control (AFTC) [3,4,5] and (2) passive fault-tolerant control (PFTC) [6,7].

In AFTC, to ensure the stability of the system, fault detection and fault diagnosis (FDD) are used to compensate with a conventional controller. The performance of AFTC highly depends on the accuracy of the FDD process. Therefore, numerous FDD methodologies have been developed to enhance the accuracy of FDD [8,9,10]. In robot manipulator systems, the FDD is typically supplanted by fault estimation (FE), a process that comprehensively estimates, detects, and isolates faults in a single step. This AFTC structure control is similar to disturbance observer-based (DOB) control approaches [11,12,13]. The key distinction between AFTC and DOB lies in the utilization of the estimated value from FE, which is compared with a predefined threshold for fault detection and isolation. Additionally, these estimated values serve to alert operators, prompting them to take efficient actions for maintenance. A notable advancement of AFTC is the ability to deal with high-magnitude faults or multi-fault scenarios. However, AFTC has a slow response to faults due to the delayed feedback from FE. The consequential effect is the occurrence of peaking phenomena, which reduce the performance of the robot system [3,14].

In contrast, PFTC uses a single controller for both normal and fault condition. This structural control approach enables the robot to respond more rapidly to faults than the AFTC scheme. Therefore, peaking phenomena are reduced in comparison with the AFTC scheme. Sliding Mode Control (SMC) stands out as a popular technique considered for PFTC. Given its robustness and ability to deal with disturbances and uncertainties, SMC has received significant attention in PFTC research [15,16,17]. In addition, the finite and fixed-time convergence are advantageous features of SMC, which have attracted the efforts of many researchers [18,19,20]. However, relying solely on the SMC technique renders the robot system less effective in addressing high-magnitude faults. The second most popular PFTC approach is adaptive control [21,22], which can handle high-magnitude faults. It quickly adjusts the control strategy in response to faults, with the capability of real-time adjustments. In addition, this technique can deliver high performance, even with less-precise dynamic robot models. Based on the analysis above, it can be seen that the choice between AFTC and PFTC depends on the model of the system and the understanding the system designer, with the potential to optimize the performance of the robot system. This paper focuses on the development of PFTC to provide a rapid response to faults and enhance the system’s ability to handle high-magnitude faults based on an adaptive technique.

To enhance the response of a robotic manipulator system to faults, the synchronization technique was used [3,18,23]. The synchronization technique was introduced in [24,25]. This method was well known in closed-loop robot systems like the dual-drive gantry mechanism [26], parallel robot manipulator [27,28], cable-driven robot manipulator [29], multi-robot cooperation [30], etc. The synchronization technique based on the cross-error was developed by Y. Koren [24] for computerized numerical control (CNC) machines. Its main idea is that dependence axes can be controlled by considering the effects of the other axes through cross-error. In general, in closed-loop mechanism systems, internal tensor force occurs during the motion of the actuator. This force can degrade the performance or damage the system. The synchronous technique causes the position error of each joint to simultaneously approach zero; therefore, it reduces the internal tensor force. In contrast, serial robots with open-loop mechanisms may not contain the internal tensor force due to the consideration of the dynamic model. Therefore, the synchronization will not provide an advantage when it is compared with model-base controllers in open-loop systems. However, the feature of each position error simultaneously approaching zero still exists when the synchronization control is applied to a serial robot. This feature will help the robot system rapidly respond to faults. Therefore, the performance of the robot will increase when faults occur in the system. Synchronous control techniques applied to fault-tolerant control of serial manipulators were first introduced in [3,18,23]. Experimental results on real robotic systems have demonstrated the unique advantages of this technique when dealing with high-magnitude faults. Despite the proven effectiveness of synchronous control, the authors have also pointed out limitations when combined with an extended state observer in AFTC schemes. Specifically, the peaking phenomenon arises when faults initially occur in the system. This happens due to the delayed response of the controller, which relies on fault information from the fault estimator. This phenomenon significantly reduces the system’s performance. Unlike the approaches in [3,18,23], this paper utilizes synchronization within the PFTC scheme to enhance the response, reduce the peaking phenomena, and improve the ability to handle high-magnitude faults. A novel synchronization control is proposed. The stability of the system is proved by Lyapunov theory.

The adaptive methodology based on intelligence techniques has received significant attention from researchers and engineers across various domains in automatic control. This approach may rely on either fuzzy logic techniques [31,32] or neural networks [33,34,35]. Fuzzy logic control typically requires the experience of an operator to design the fuzzy rules, while neural networks eliminate the operator’s need for specific knowledge of network design. Two types of popular neural network architectures in controllers are feed-forward networks and recurrent networks. However, these kinds of neural networks have some drawbacks such as local minima, slow convergence, and challenges in selecting learning rate and initial weight parameters. To address these limitations, researchers have proposed various solutions, as seen in [36,37], where the authors provided a technique for selecting initial weights and improving the convergence time. Another strategy to enhance the convergence time and overcome the local minimum is the use of a momentum part, as suggested in [38,39]. In addition, orthogonal neural networks (ONNs) were presented by Yen and Chen in [40] to overcome the drawbacks of traditional neural network architectures. This kind of neural network has been applied to deep neural networks [41,42,43] and shown to be effective in comparison to feed-forward networks. In automatic control, ONNs have been applied to anti-lock braking systems [44] and robot control [28,45]. In this paper, the ONN is designed to increase the ability to manage high-magnitude faults and provide fast responses to faults based on the online compensation of the ONN.

In this paper, an adaptive fault-tolerant control for multi-joint robot manipulators based on a combination of synchronous techniques and neural networks is proposed to enhance performance. Initially, the PFTC synchronous terminal sliding mode controller (PFTC S-TSMC) is investigated by exploiting the synchronization error with a novel synchronous sliding surface. Subsequently, an adaptive fault-tolerant control based on the combination of synchronization controller and ONN (PFTC NN-S-TSMC) is put forward to improve the ability to manage high-magnitude faults. Finally, both proposed PFTC controllers are verified on a 3-DOF robot manipulator using MATLAB Simulink to demonstrate the advantages of the proposed controller. The contributions of this paper can be summarized as follows:

(1) A passive fault-tolerant control scheme based on a novel robust synchronous terminal sliding mode control is designed. By simultaneously driving each joint position error to zero using a synchronization technique, the system exhibits a rapid response to faults. This approach mitigates the peaking phenomenon typically seen in active fault-tolerant control methods, which often suffer from slow response times [18]. The proposed controller demonstrates a faster response to faults and is more effective in handling high-magnitude faults compared to conventional terminal sliding mode control techniques [46]. The enhanced performance of the fault-tolerant control is attributed to the incorporation of additional joint information, which further improves the system’s effectiveness.

(2) An adaptive fault-tolerant control based on the neural network synchronous terminal sliding mode is proposed. Due to the combination of the advantages of the synchronization technique and online adjustment of the neural network, the proposed controller outperforms the robust proposed controller and conventional terminal sliding mode controller [46]. By using the orthogonal neural network, the advantages in low-level control systems are shown. Unlike in [47], using the position error, the input to the orthogonal neural network in the proposed controller is the synchronization error, ensuring that the synchronization feature is maintained during the neural network’s online weight updates.

(3) The stability of the system is proven with Lyapunov theory for both of the proposed controllers.

The rest of this paper is organized as follows. In Section 2, the dynamic model of the robot, faults, and orthogonal neural network are given. The novel synchronization sliding surface, proposed robust synchronous terminal sliding mode control, and an adaptive fault-tolerant control based on synchronization and orthogonal neural networks are proposed in Section 3. In Section 4, a simulation of the proposed control in a 3-DOF robot manipulator using MATLAB Simulink is presented. A discussion of the limitations and future work is provided in Section 5. Finally, the conclusions are given in Section 6.

## 2. Preliminaries

### 2.1. Dynamic Model of Robot Manipulators

The dynamic model of an *n*-degree robot manipulator is governed by the following equation [48]:

(1) $$M\left(q\right)\ddot{q}+C\left(q,\dot{q}\right)\dot{q}+G\left(q\right)+\Xi =\tau$$

where $\ddot{q},\dot{q},q\in {ℜ}^{n}$ are the vectors of joint acceleration, velocity, and position, respectively. $M\left(q\right)\in {ℜ}^{n\times n}$, $C\left(q,\dot{q}\right)\in {ℜ}^{n}$, and $G\left(q\right)\in {ℜ}^{n}$ represent the inertia matrix, the centripetal and Coriolis matrix, and the gravitation force, respectively. $\tau \in {ℜ}^{n}$ is the torque at the joints. $\Xi \in {ℜ}^{n}$ represents the unknown dynamic uncertainties and external disturbances.

**Assumption 1.**  *The matrices M(q) and *Ξ* are bounded by the following:*

$$\begin{array}{c} M_{m}\leq ∥M^{-1}\left(q\right)∥\leq M_{M}\\ ||\Xi ||<a_{0}+a_{1}\left||q\left(t\right)|\right|+a_{2}\left|\right|\dot{q}{\left(t\right)||}^{2}+\gamma ||\tau || \end{array}$$

*where $M_{m}$, $M_{M}$, and γ are known as positive constants.*

### 2.2. Model of Joint Actuator Faults

In robot manipulator systems, an actuator joint consists of many electrical and mechanical components such as an electronic motor, motor driver, gear, etc. Therefore, the occurrence of faults/failure at an actuator joint has a high probability. For example, a decrease in motor torque can result from increased friction of the stator and robot inside the electric motor after being in use for a long time, or it can result from increased friction of a mechanical part due to the environment such as temperature or dust. It can be considered as a loss-effectiveness fault. Generally, faults in robot manipulator systems can be represented as shown in Equation (2), where two common faults are loss-effectiveness and bias faults [49]:

(2) $$\tau^{t}=\left(I-\rho \left(t\right)\right)\tau +f\left(t\right)\;\;\;\;\left(t>t_{f}\right)$$

where $\tau^{t}\in {ℜ}^{n}$ is the vector of torques at the output joint actuator. $\tau \in {ℜ}^{n}$ is the vector of torques at the output controller. ρ(t)$=diag\left(\rho_{i}\left(t\right)\right)\in {ℜ}^{n\times n},\;0\leq \rho_{i}\left(t\right)<1,\;i=\left(1,2,\ldots ,n\right)$ denotes the loss-effective rate. $f\left(t\right)\in {ℜ}^{n}$ is the vector of the bias fault. $t_{f}$ is the time of the occurrence of faults. $I\in {ℜ}^{n\times n}$ is an identity matrix.

Substituting Equation (2) into Equation (1), the dynamic model of an *n*-DOF robot manipulator with actuator faults can be written as follows:

(3) $$M\left(q\right)\ddot{q}+C\left(q,\dot{q}\right)\dot{q}+G\left(q\right)+\Xi =\left(I-\rho \left(t\right)\right)\tau +f\left(t\right)$$

In this paper, the investigation is focused on the effects of loss-effective and bias faults in robot manipulators. In loss-effective faults, when $\rho_{i}\left(t\right)=1$, they are considered as lock-in-place faults, which are not investigated because the robot manipulator in this paper does not have a redundancy actuator. Therefore, the robot cannot tolerate this type of fault.

### 2.3. Orthogonal Neural Network

In this section, the conventional ONN is given. It is based on a feed-forward neural network (FNN) and polynomial functions as activity functions.

According to the theory of orthogonal function [50], an arbitrary function will have an orthogonal polynomial as follows:

(4) $$\begin{array}{c} F_{n}\left(x\right)=w_{1}\lambda_{1}\left(x\right)+w_{2}\lambda_{2}\left(x\right)+\ldots +w_{n}\lambda_{n}\left(x\right) \end{array}$$

such that

(5) $$\begin{array}{c} \lim_{n\to \infty }\int_{a}^{b}{\left(f\left(x\right)-F_{n}\left(x\right)\right)}^{2}dx=0 \end{array}$$

where

(6) $$\begin{array}{c} \int_{a}^{b}\lambda_{i}\left(x\right)\lambda_{j}\left(x\right)dx=\left\{\begin{array}{cc} 0, & \;\mathrm{if}\;i\neq j\\ A_{j}, & \;\mathrm{if}\;i=j \end{array}\right. \end{array}$$

(7) $$\begin{array}{c} w_{i}=\int_{a}^{b}f\left(x\right)\lambda_{i}\left(x\right)dx/A_{i}\left(i=1,2,\ldots ,n\right) \end{array}$$

in which $\left\{\lambda_{1}\left(x\right),\lambda_{2}\left(x\right),\ldots \right\}$ is an orthogonal set. The orthogonal functions include Fourier series, the Bessel function, and Legendre and Chebyshev polynomials.

In the case where there is a function with *m* variables, an orthogonal function set is denoted as $\left\{\Psi_{1}\left(x\right),\Psi_{2}\left(x\right),\ldots \right\}$. Then, each orthogonal function is defined as follows:

(8) $$\begin{array}{c} \Psi_{i}\left(X\right)=\lambda_{1i}\left(x_{1}\right)\lambda_{2i}\left(x_{2}\right)\ldots \lambda_{mi}\left(x_{n}\right) \end{array}$$

where $X={\left[x_{1},x_{2},\ldots ,x_{n}\right]}^{T}$ is the input vector.

**Figure 1:**
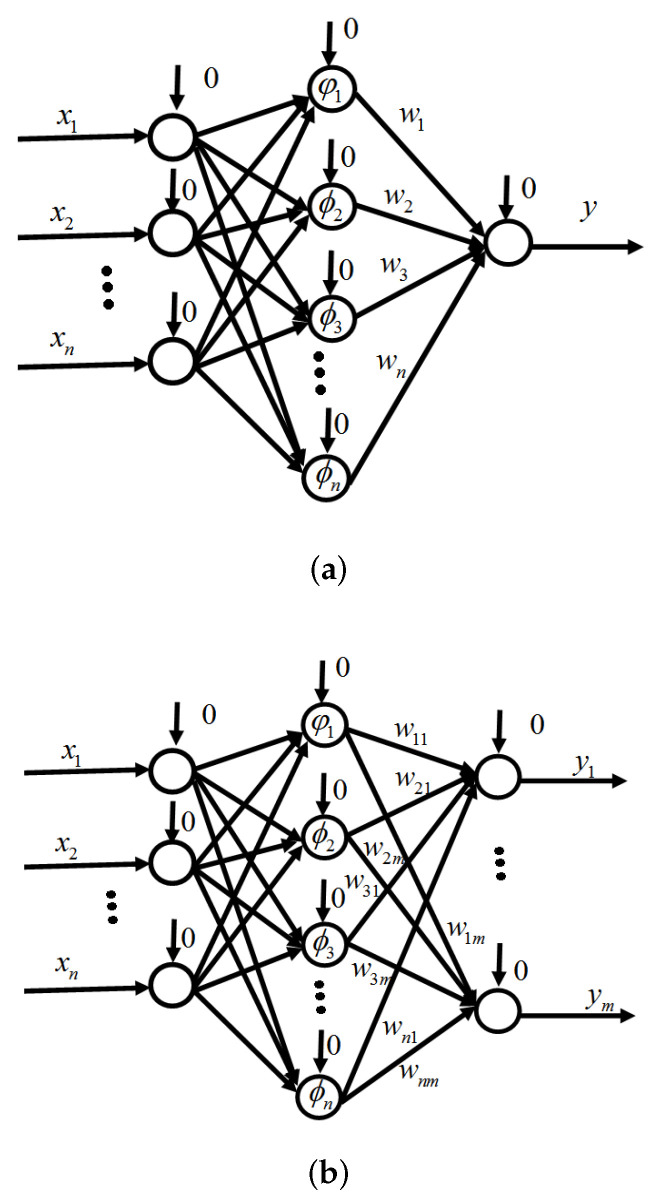
The orthogonal neural network. (**a**) Single output, (**b**) multiple outputs.

An ONN based on a feed-forward network with one hidden layer is shown in Figure 1 by [40]. Figure 1 shows (a) multi input–one output and (b) multi input–multi output. The weight connections between the input layer and hidden layer are 1, and the bias is 0. $\lambda_{i}\left(i=0,1,2,\ldots ,m\right)$ is an orthogonal function. The weights between them are $w_{ij}$ (where *i* is number of orthogonal functions and *j* is number of outputs). The output of the ONN [40] can be shown as follows:

(9) $$\begin{array}{c} y=F\left(X\right)=\sum_{i=1}^{n}w_{i}\left(t\right)\Psi_{i}\left(X\right)=W^{T}\left(t\right)\Psi \end{array}$$

### 2.4. Definitions

Some definitions are necessary to propose the fault-tolerant control law.

**Definition 1.**  *We define ${\left\lceil x\right\rfloor }^{\Lambda }=\left[|x_{1}{|}^{\lambda_{1}}sign\left(x_{1}\right),|x_{2}{|}^{\lambda_{2}}sign\left(x_{2}\right),\;\ldots ,{\left|x_{n}\right|}^{\lambda_{n}}sign\left(x_{n}\right)\right]\in {ℜ}^{n}$, where $\lambda_{i}>0$ and $\Lambda =diag(\lambda_{i})$ with (i=1,2,…,n). $x={\left[x_{1},x_{2},\ldots ,x_{n}\right]}^{T}\in {ℜ}^{n}$. $y={\left[y_{1},y_{2},\ldots ,y_{n}\right]}^{T}\in {ℜ}^{n}$ and sign(x) is a signum function, which is defined as follows:*

(10) $$sign\left(x\right)=\left\{\begin{array}{c} -1,\;if\;\;x<0\\ 0,\;if\;\;x=0\\ 1,\;if\;\;x>0 \end{array}\right.$$

**Definition 2.**  *We define $x\cdot y=\left[x_{1}y_{1},x_{2}y_{2},\ldots ,x_{n}y_{n}\right]\in {ℜ}^{n}$.*

**Definition 3.**  *The time derivative of ${\left\lceil x\right\rfloor }^{\Lambda }$ is $\frac{d}{dt}{\left\lceil x\right\rfloor }^{\Lambda }={\Lambda |x|}^{\Lambda -1}\cdot x=\left[\lambda \right|x_{1}{|}^{\lambda -1}x_{1},\lambda {\left|x_{2}\right|}^{\lambda -2}x_{2},$ …, $\lambda |x_{n}{{|}^{\lambda -n}x_{n}]}^{I}$ and $x^{\Lambda -1}=\left[x_{1}^{\lambda_{1}-1},x_{2}^{\lambda_{2}-1},\ldots ,x_{n}^{\lambda_{n}-1}\right]\in {ℜ}^{n}$ where $I=diag\left(1\right)\in {ℜ}^{n\times n}$.*

## 3. Fault-Tolerant Control with Synchronous Terminal Sliding Mode Control

In this part, passive fault-tolerant control based on the synchronous terminal sliding mode control is proposed.

### 3.1. Synchronization Error

A synchronization error [18] is given as follows:

(11) $$\left\{\begin{array}{c} \varepsilon_{1}=\left(1+\psi_{1}\psi_{n}\right)e_{1}-\psi_{1}e_{2}-\psi_{1}e_{n}\\ \varepsilon_{2}=\left(1+\psi_{2}\psi_{1}\right)e_{2}-\psi_{2}e_{3}-\psi_{2}e_{1}\\ \;\;\;\;\;\vdots \\ \varepsilon_{n}=\left(1+\psi_{n}\psi_{n-1}\right)e_{n}-\psi_{n}e_{2}-\psi_{n}e_{1} \end{array}\right.$$

where $e_{i}=q_{di}-q_{i}\;\left(i=1,2,\ldots ,n\right)$ is the error at each joint, where $q_{di}$ and $q_{i}$ are the desired position and current position of the joint *i*, respectively. $\psi_{i}\left(i=1,2,\ldots ,n\right)$ is the corresponding positive gain. In matrix form,

(12) ε=Te

where $\varepsilon ={\left[\varepsilon_{1},\varepsilon_{2},\ldots ,\varepsilon_{n}\right]}^{T}\in {ℜ}^{n}$, $e={\left[e_{1},e_{2},\ldots ,e_{n}\right]}^{T}\in {ℜ}^{n}$, $T\in {ℜ}^{n\times n}$ is a transformation matrix, and

(13) $$T=\left[\begin{array}{ccccc} \left(1+\psi_{1}\psi_{n}\right) & -\psi_{2} & 0 & \cdots & -\psi_{n}\\ -\psi_{1} & \left(1+\psi_{2}\psi_{1}\right) & -\psi_{3} & \cdots & 0\\ 0 & -\psi_{2} & \left(1+\psi_{3}\psi_{2}\right) & \cdots & 0\\ \vdots & \vdots & \vdots & \ddots & \vdots \\ -\psi_{1} & 0 & 0 & \cdots & \left(1+\psi_{n}\psi_{n-1}\right) \end{array}\right]$$

### 3.2. Proposed Robust Fault-Tolerant Control with Synchronization Error

In this section, robust fault-tolerant control with synchronization error based on terminal sliding mode control is proposed.

Equation (3) can be rewritten as follows:

(14) $$\ddot{q}=M{\left(q\right)}^{-1}\left(\tau -H\left(q,\dot{q}\right)-\zeta \right)$$

where $H\left(q,\dot{q}\right)=C\left(q,\dot{q}\right)\dot{q}+G\left(q\right)$, ζ=ρ(t)τ+Ξ−f(t) represents the uncertainties, disturbances, and fault.

The proposed synchronous fast terminal sliding mode surface based on novel synchronous error is given as follows:

(15) $$S=\dot{e}+\beta e+\alpha {\left\lceil \varepsilon \right\rfloor }^{\Lambda }$$

where $S=\left[s_{1},s_{2},s_{2},\ldots ,s_{n}\right]\in {ℜ}^{n}$ is the vector of the sliding surface. $\alpha =diag\left(\alpha_{i}\right)\in {ℜ}^{n\times n}$, $\beta =diag\left(\beta_{i}\right)\in {ℜ}^{n\times n}$, and $\Lambda =diag\left(\lambda_{i}\right)\in {ℜ}^{n\times n}$ are the positive matrix gain, with 0≤λ≤1 and i=1,2,…,n.

From the novel synchronous sliding surface, the proposed passive fault-tolerant control with synchronous terminal sliding mode (PFTC S-TSMC) is given as follows:

(16) $$\tau =\tau_{0}+\tau_{1}+\tau_{2}$$

where

(17) $$\tau_{0}=M\left(q\right)\ddot{q_{d}}+H\left(q,\dot{q}\right)$$

(18) $$\tau_{1}={M\left(q\right)(\alpha \Lambda |\varepsilon |}^{\Lambda -1}\dot{\varepsilon }+\beta \dot{e}+Ksign\left(S\right))$$

(19) $$\tau_{2}=\left\{\begin{array}{c} \frac{S}{\left||S|\right|}w,\;||S||\neq 0,\;w=\frac{1}{1-\gamma }(a_{0}+a_{1}\left||q|\right|+a_{2}\left|\right|\dot{q}{\left|\right|}^{2}+\gamma ||\tau_{0}+\tau_{1}\left||\right)\\ 0,\;||S||=0 \end{array}\right.$$

where $\alpha =diag\left(\alpha_{i}\right)\in {ℜ}_{n\times n}$, $\beta =diag\left(\beta_{i}\right)\in {ℜ}_{n\times n}$, $K=diag\left(k_{i}\right)\in {ℜ}_{n\times n}$, $a_{0}$, $a_{1}$, $a_{2}$, and γ are positive constants, and γ is given as follows:

(20) $$\gamma =\frac{M_{M}-M_{m}}{M_{M}+M_{m}},$$

**Theorem 1.**  *Given the uncertain robot manipulator system in Equation (3) and the proposed controller in Equation (16) and based on the novel synchronization error in Equation (12) and sliding surface in Equation (15), the tracking trajectory error of the robot manipulator converges to zero.*

**Proof of Theorem 1.**  The Lyapunov function can be selected as follows:

(21) $$V=\frac{1}{2}S^{T}S,$$

The time derivative of V in Equation (21) is as follows:

(22) $$\begin{array}{cc} \dot{V} & =S^{T}\dot{S}\\ \; & =S^{T}(\ddot{e}+{\alpha \Lambda |\varepsilon |}^{\Lambda -1}\dot{\varepsilon }+\beta \dot{e})\\ \; & =S^{T}(\ddot{q_{d}}-\ddot{q}+{\alpha \Lambda |\varepsilon |}^{\Lambda -1}\dot{\varepsilon }+\beta \dot{e}) \end{array}$$

Substituting Equation (14) into Equation (22) we have the following:

(23) $$\dot{V}=S^{T}(\ddot{q_{d}}-\left(M{\left(q\right)}^{-1}\left(\tau -H\left(q,\dot{q}\right)-\zeta \right)\right)+{\alpha \Lambda |\varepsilon |}^{\Lambda -1}\dot{\varepsilon }+\beta \dot{e})$$

Substituting the proposed controller in Equation (16) into Equation (23), we obtain the following:

(24) $$\dot{V}=-S^{T}M^{-1}\left(q\right)\left(\frac{S}{\left||S|\right|}w-\zeta \right)-S^{T}Ksign\left(S\right)$$

We can see that $\left||\tau |\right|\leq \left|\right|\tau_{0}+\tau_{1}\left|\right|+\left|\right|\tau_{2}\left|\right|$, and applying Assumption 1 yields the following:

(25) $$\begin{array}{cc} \dot{V} & \leq -S^{T}M_{m}(\frac{S}{\left||S|\right|}w-(a_{0}+a_{1}\left||q\left(t\right)|\right|+a_{2}\left|\right|\dot{q}{\left(t\right)||}^{2}+\gamma ||\tau ||))-S^{T}Ksign\left(S\right)\\ & \leq -S^{T}M_{m}(\frac{S}{\left||S|\right|}w-(a_{0}+a_{1}\left||q\left(t\right)|\right|+a_{2}\left|\right|\dot{q}{\left(t\right)||}^{2}+\gamma ||\tau_{0}+\tau_{1}\left|\right|+\gamma ||\tau_{2}\left||)\right)-S^{T}Ksign\left(S\right)\\ & =M_{m}(-||S||w+||S||(a_{0}+a_{1}\left||q\left(t\right)|\right|+a_{2}\left|\right|\dot{q}{\left(t\right)||}^{2}+\gamma ||\tau_{0}+\tau_{1}\left||\right)+\gamma ||\tau_{2}\left||\right)-S^{T}Ksign\left(S\right)\\ & =M_{m}\left(-||S|\right|\frac{1}{1-\gamma }w+||S||w+\gamma ||\tau_{2}\left||||S||\right)-S^{T}Ksign\left(S\right)\\ & =M_{m}\left(\gamma |\right|\tau_{2}\left||||S|\right|-\frac{\gamma }{1-\gamma }\left||S||w\right)-S^{T}Ksign\left(S\right) \end{array}$$

Substituting Equation (18) into Equation (25), we have the following:

(26) $$\dot{V}\leq -S^{T}Ksign\left(S\right)\leq -\sigma V^{\frac{1}{2}}\leq 0$$

where $\sigma =\lambda_{min}K$. When the sliding surface converges to zero, we have the position error $e_{i}\to 0$. Therefore, Theorem 1 is proven. □

### 3.3. Proposed Neural Network-Based Fault-Tolerant Control with Synchronization Error

In this section, the proposed fault-tolerant control neural network-based synchronization error (PFTC NN-S-TSMC) is presented.

The ONN is adaptively used to estimate the lump of uncertainties, disturbances, and faults ζ in Equation (13).

(27) $$\zeta =W^{T}\Psi \left(X\right)+\epsilon$$

$X\in {ℜ}^{n}$ is the input of the network. ϵ is the approximation error of the neural network. The Chebyshev polynomial is used as an orthogonal function Ψ(X). The ONN in this proposed controller has three layers:

1. The input layer:
  The input vector is denoted as follows:
  (28) $$X=x_{i}^{T}={\left[\varepsilon_{i},{\dot{\varepsilon }}_{i}\right]}^{T}\;\left(i=\left(1,2,\ldots ,n\right)\right)$$
  where $\varepsilon_{i}$ and $\dot{\varepsilon_{i}}$ are the synchronization error and synchronization velocity, respectively. Due to the condition of the ONN, the input must be within the range of [1,−1] so that the input can be transformed as follows:
  (29) $$x_{i}=\frac{2}{b-a}x_{i}-\frac{b-1}{b-a}$$
  where *a* and *b* are the lower and upper bounds of $x_{i}$, respectively.
2. The hidden layer:
  The Chebyshev polynomial used as an orthogonal function Ψ(X) is shown as follows:
  (30) $$\lambda_{i}\left(x\right)=\cos\left(n\arccos(x)\right)$$
  It can be rewritten as follows:
  (31) $$\left\{\begin{array}{c} \lambda_{0}=1\\ \lambda_{1}=x\\ \lambda_{2}=2x^{2}-1\\ \lambda_{3}=4x^{3}-3x\\ \;\;\;\;\;\vdots \\ \lambda_{m+1}=2x\lambda_{m}\left(x\right)-\lambda_{m-1}\left(x\right) \end{array}\right.$$
3. The output layer:
  The output of the ONN is expressed as follows:
  (32) $$y=W^{T}\Psi \left(X\right)$$
  where Ψ(X) is shown in Equation (7).

The proposed weight update law is given as follows:

(33) $$\dot{\hat{W}}=-\eta S^{T}M^{-1}\left(q\right)\Psi \left(X\right)$$

where η is the learning rate. The proposed controller based on ONN is given as follows:

(34) $$\tau =\tau_{0}+\tau_{1}+\tau_{2}$$

where

(35) $$\tau_{0}=M\left(q\right)\ddot{q_{d}}+H\left(q,\dot{q}\right)$$

(36) $$\tau_{1}={M\left(q\right)(\alpha \Lambda |\varepsilon |}^{\Lambda -1}\dot{\varepsilon }+\beta \dot{e}+Ksign\left(S\right))$$

(37) $$\tau_{2}={\hat{W}}^{T}\Psi \left(X\right)$$

where $\hat{W}$ is the approximation of *W* in Equation (28).

**Theorem 2.**  *Given the uncertain robot manipulator system in Equation (3) and the proposed controller in Equation (34) and based on the novel synchronization error in Equation (12) and sliding surface in Equation (15) with the orthogonal neural network using the weight update law in Equation (34), the tracking trajectory error of the robot manipulator converges to zero.*

**Proof of Theorem 2.**  The Lyapunov function can be selected as follows:

(38) $$V=\frac{1}{2}S^{T}S+\frac{1}{2}\eta {\tilde{W}}^{T}\tilde{W}$$

where $\tilde{W}$ is the error approximation of *W*. It can be shown as follows:

(39) $$\begin{array}{cc} \tilde{\zeta } & =\zeta -\hat{\zeta }\\ \; & =W^{T}\Psi \left(X\right)+\epsilon -{\hat{W}}^{T}\Psi \left(X\right)\\ \; & ={\tilde{W}}^{T}\Psi \left(X\right) \end{array}$$

The time derivative of V in Equation (39) is as follows:

(40) $$\begin{array}{cc} \dot{V} & =S^{T}\dot{S}+\eta {\tilde{W}}^{T}\dot{\tilde{W}}\\ \; & =S^{T}(\ddot{e}+{\alpha \Lambda |\varepsilon |}^{\Lambda -1}\dot{\varepsilon }+\beta \dot{e})+\eta {\tilde{W}}^{T}\dot{\tilde{W}}\\ \; & =S^{T}(\ddot{q_{d}}-\ddot{q}+{\alpha \Lambda |\varepsilon |}^{\Lambda -1}\dot{\varepsilon }+\beta \dot{e})+\eta {\tilde{W}}^{T}\dot{\tilde{W}} \end{array}$$

Substituting Equation (14) into Equation (39), we have the following:

(41) $$\dot{V}=S^{T}(\ddot{q_{d}}-\left(M{\left(q\right)}^{-1}\left(\tau -H\left(q,\dot{q}\right)-\zeta \right)\right)+{\alpha \Lambda |\varepsilon |}^{\Lambda -1}\dot{\varepsilon }+\beta \dot{e})+\eta {\tilde{W}}^{T}\dot{\tilde{W}}$$

Substituting the proposed controller in Equation (34) into Equation (41), we obtain the following:

(42) $$\dot{V}=S^{T}\left(-M^{-1}\left(q\right)\left({\hat{W}}^{T}\Psi \left(X\right)-\zeta \right)-Ksign\left(S\right)\right)+\eta {\tilde{W}}^{T}\dot{\tilde{W}}$$

Substituting Equation (28) into Equation (42) yields the following:

(43) $$\begin{array}{cc} \dot{V} & =S^{T}M^{-1}\left(q\right)\left({\tilde{W}}^{T}\Psi \left(X\right)+\epsilon \right)-Ksign\left(S\right))+\eta {\tilde{W}}^{T}\dot{\tilde{W}}\\ \; & ={\tilde{W}}^{T}\left(S^{T}M^{-1}\left(q\right)\Psi \left(X\right)+\eta \dot{\tilde{W}}\right)+S^{T}M^{-1}\left(q\right)\epsilon -S^{T}Ksign\left(S\right) \end{array}$$

Substituting the update law in Equation (33) into Equation (43), we obtain the following:

(44) $$\begin{array}{cc} \dot{V} & =S^{T}M^{-1}\left(q\right)\epsilon -KS^{T}sign\left(S\right))\\ \; & \leq -K||S||+S^{T}M_{M}\epsilon \end{array}$$

$\dot{V}\leq 0$ approximately when the design parameter $K\geq M_{M}\epsilon$ with the approximation error ϵ is sufficiently small. Therefore, Theorem 2 is proven. □

## 4. Simulation Results

### 4.1. Simulation Setup

**Figure 2:**
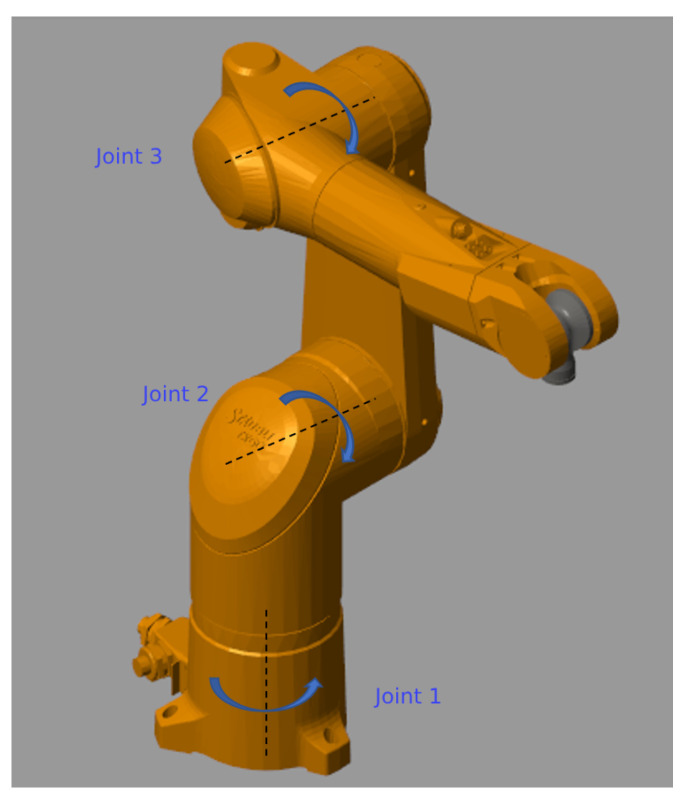
Three-degrees-of-freedom (DOF) Staubli TX60L robot manipulator in MATLAB/Simulink with active joints.

In this section, we present and discuss the simulation results for a conventional terminal sliding mode control and the two proposed FTC S-TSMC and FTC NN-S-TSMC controllers after applying them to a 3-DOF robot manipulator. The mechanical model of the 3-DOF robot manipulator is constructed using SolidWorks 2024 software. The geometry parameters were taken from the Staubli TX60L robot manipulator [51]. To reduce the complexity of the dynamic model calculations of the robot, joints 4, 5, and 6 of the TX60L were disabled in this study. Subsequently, the robot manipulator model was exported to a MATLAB simulation environment using the Simmechanics toolbox, as illustrated in Figure 2. The detailed parameters of the robot manipulator can be found in Table 1.

For this trajectory-tracking simulation, the desired trajectories at each joint are given as follows:

(45) $$\left\{\begin{array}{c} q_{1d}=0.5cos\left(t/2\right)-0.5\\ q_{2d}=0.3cos\left(t/2\right)-0.3\\ q_{3d}=0.2cos\left(t/2\right)-0.2 \end{array}\right.$$

where $\dot{q_{d}}$ and $\ddot{q_{d}}$ are the first-order and second-order derivatives of the desired position, respectively.

The uncertainties at each joint are assumed to be as follows:

(46) $$\left\{\begin{array}{c} \Xi_{1}=0.2sign\left(q_{1}\right)+0.3\dot{q_{1}}\\ \Xi_{2}=0.3sign\left(q_{2}\right)+0.3\dot{q_{2}}\\ \Xi_{3}=0.32sign\left(q_{3}\right)+0.2\dot{q_{3}} \end{array}\right.$$

The parameters in the two proposed controllers in Equations (16) and (34) are selected as $\psi_{1}=\psi_{2}=\psi_{3}=2$, α=diag(0.5,0.5,0.5), β=diag(0.72,0.72,0.72), Λ=diag(0.6,0.6,0.6), K=diag(80,80,110), γ=0.2, $a_{0}=5$, $a_{1}=20$, $a_{2}=5$, η=0.01.

The conventional fast terminal sliding mode control (FTSMC) [46] is given as follows:

(47) $$\tau =\tau_{0}+\tau_{1}$$

where $\tau_{0}=M\left(q\right)\left({\ddot{q}}_{d}+d\dot{e}+c\Lambda e^{\Lambda -1}\dot{e}\right)+H\left(q,\dot{q}\right)$, $\tau_{1}=M\left(q\right)K_{1}sign\left(S\right)$.

The conventional fast terminal sliding surface [46] is selected as follows:

(48) $$S=\dot{e}+de+ce^{\Lambda }$$

The parameters for the conventional FTSMC are suitably chosen as c=diag(0.5;0.5;0.5), d=diag(0.72;0.72;0.72), $K_{1}=diag\left(80;80;110\right)$, Λ=diag(0.6;0.6;0.6).

The active fault-tolerant control with the synchronous terminal sliding mode in [18] (AFTC S-TSMC [18]) is described as follows. The coupling position error is given as follows:

(49) $$E=\alpha e+\beta \int {\left\lceil \epsilon \right\rceil }^{\Lambda }\;dt$$

where $E={\left[E_{1},E_{2},\cdots ,E_{n}\right]}^{T}\in {ℜ}^{n}$, $\alpha =\mathrm{diag}\left(\alpha_{i}\right)\in {ℜ}^{n\times n}$, $\beta =\mathrm{diag}\left(\beta_{i}\right)\in {ℜ}^{n\times n}$ are positive coupling parameters, and $\Lambda =\mathrm{diag}\left(\lambda_{i}\right),\;0<\lambda_{i}<1$.

The synchronous terminal sliding surface is given as follows:

(50) $$S=\dot{E}+\Gamma {\left\lceil E\right\rceil }^{\Lambda }$$

where $S={\left[S_{1},S_{2},\cdots ,S_{n}\right]}^{T}\in {ℜ}^{n}$, $\dot{E}={\left[{\dot{E}}_{1},{\dot{E}}_{2},\cdots ,{\dot{E}}_{n}\right]}^{T}\in {ℜ}^{n}$, $\Gamma =\mathrm{diag}\left(\gamma_{i}\right)\in {ℜ}^{n\times n}$, $\gamma_{i}>0$. AFTC S-TSMC [18] is given as follows:

(51) $$\tau =\tau_{eq}+\tau_{0}+\tau_{ob}$$

where $\tau_{eq}=M\left(q\right)\left({\ddot{q}}_{d}+\alpha^{-1}{\beta \Lambda |\epsilon |}^{\Lambda -I}\cdot \dot{\epsilon }+\alpha^{-1}\Gamma \Lambda {\left|\dot{\epsilon }\right|}^{\Lambda -I}\cdot \dot{E}\right)+H\left(q,\dot{q}\right)$, $\tau_{0}=M\left(q\right)K_{1}sign\left(S\right)$, $\tau_{ob}=-M\left(q\right)\hat{\phi }$ which $K_{1}=diag\left(k_{1i}\right)\in R^{n\times n}$. We obtain $\hat{\phi }$ as follows:

(52) $$\left\{\begin{array}{cc} {\hat{x}}_{1} & ={\hat{x}}_{2}+\frac{\alpha_{1}}{\epsilon }\left(x_{1}-{\hat{x}}_{1}\right)\\ {\hat{x}}_{2} & =\hat{f}\left(x_{1},{\hat{x}}_{2},\tau \right)+\frac{\alpha_{2}}{\epsilon^{2}}\left(x_{1}-{\hat{x}}_{1}\right)+\hat{\phi }\\ \hat{\phi } & =\frac{\alpha_{1}}{\epsilon^{3}}\left(x_{1}-{\hat{x}}_{1}\right) \end{array}\right.$$

where ${\hat{x}}_{1},{\hat{x}}_{2},\hat{f},\;\mathrm{and}\;\hat{\phi }$ are the estimates of $x_{1}=q\in {ℜ}^{n},x_{2}=\dot{q}\in {ℜ}^{n},f=M^{-1}\left(q\right)\left(\tau -H\left(q,\dot{q}\right)\right),\;\mathrm{and}\;\phi =-M^{-1}\left(q\right)\zeta$, respectively, $\alpha_{1},\alpha_{2},\alpha_{3}$ are positive constants, and 0<ϵ<1. The parameters for the AFTC S-TSMC [18] were obtained as follows: $\psi_{1}=\psi_{2}=\psi_{3}=2$, α=diag(1;1;1), β=diag(0.5;0.5;0.5), Λ=diag(0.6;0.6;0.6), Γ=diag(7;7;7), and $K_{1}=\mathrm{diag}\left(80;80;110\right)$.

**Remark 1.**  *To avoid a singularity, the terms containing power Λ−I in Equations (16), (34), (47), and (55) are replaced with the saturation function.*

(53) $$sat\left(u_{f},u_{s}\right)=\left\{\begin{array}{cc} u_{s} & if\;u_{f}\geq u_{s}\\ u_{f} & if\;u_{f}<u_{s} \end{array}\right.$$

*where $u_{s}=0.3$ is a positive constant, and $u_{f}=\Lambda {\left|x\right|}^{\Lambda }-I\cdot \dot{x}\;\mathrm{with}\;x=\varepsilon$.*

**Remark 2.**  *To avoid chattering, the signum functions in Equations (16), (34), (47), and (55) are replaced with the saturation function:*

(54) $$sat\left(s\right)=\left\{\begin{array}{cc} sgn(s)\delta & if\;|s|\geq \delta \\ s & if\;|s|<\delta \end{array}\right.$$

*where δ=0.8.*

### 4.2. Simulation Results

In this subsection, the two kinds of actuator faults occurring at joint 2 are considered. The assumed fault function is shown as follows:

(55) $$\left\{\begin{array}{cc} \tau_{t1}=\tau_{1}\\ \tau_{t2}=\left(1-\rho_{2}\left(t\right)\right)\tau_{2}+f_{2}\left(t\right) & \mathrm{if}\;t\geq 5\\ \tau_{t3}=\tau_{3} \end{array}\right.$$

where $\rho_{2}\left(t\right)=0.5\sin\left(\pi t\right)$, $f_{2}\left(t\right)=80\sin\left(\frac{\pi (t-5)}{2}\right)$.

**Figure 3:**
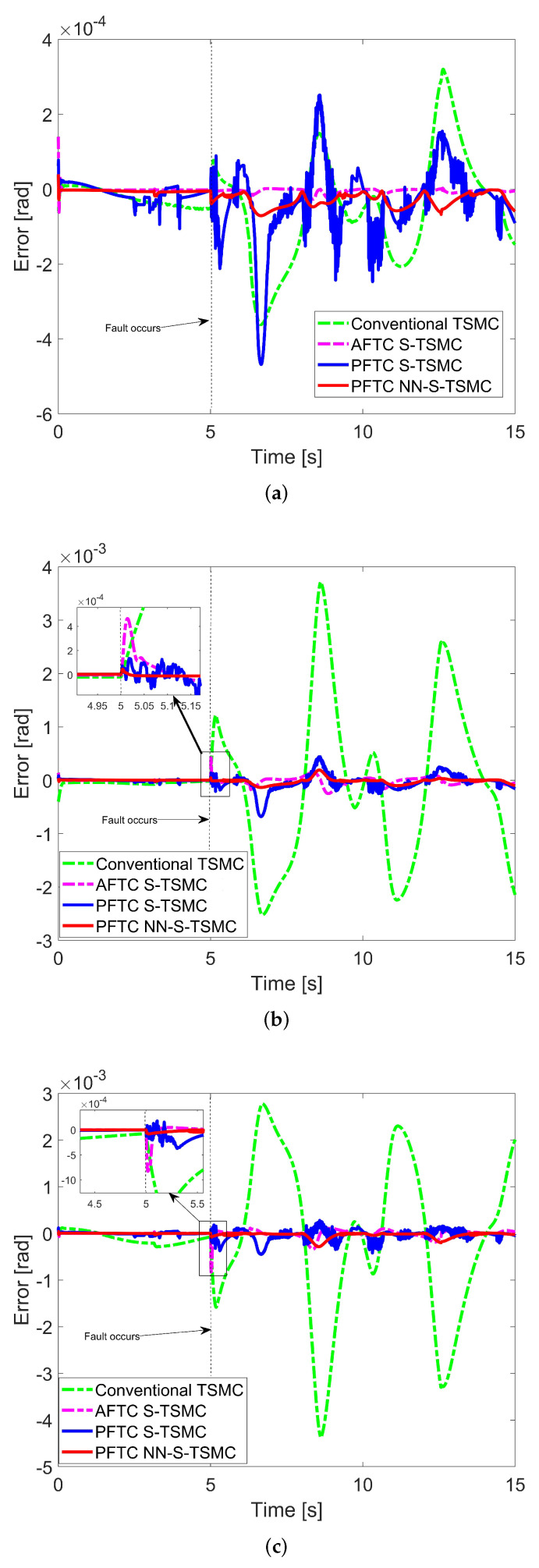
Tracking trajectory errors [18]. (**a**) Joint 1, (**b**) joint 2, (**c**) joint 3.

The results in Table 2 illustrate the effectiveness of the two proposed PFTC approaches. It is evident that the PFTC NN-S-TSMC significantly outperforms the two robust PFTCs. This superior performance can primarily be attributed to the online adaptability afforded by the neural network. Additionally, the AFTC S-TSMC [18] achieves a comparable performance level to that of the proposed PFTC NN-S-TSMC. This capability arises from its effective fault estimation processes, which enable the system to compensate for faults and maintain operational efficiency. However, despite AFTC S-TSMC’s competence, PFTC NN-S-TSMC retains distinct advantages over it, particularly in its ability to reduce the peaking phenomena due to the use of the PFTC scheme. This advantage can be seen in detail in Figure 3.

The tracking trajectory results are illustrated in Figure 3. In Figure 3a, the error levels of the four controllers are generally similar, remaining within $10^{-4}$ radians. This joint is less affected by faults due to gravity. Notably, AFTC S-TSMC [18] exhibits superior performance compared to the other controllers due to the robustness of its fault estimation. In Figure 3b,c, prior to the fault occurrence, the position errors of all four controllers are comparable. However, after the fault occurs at the 5th second, joints 2 and 3 are significantly impacted due to both the fault and the effects of gravity on joint 3. The conventional TSMC maintains acceptable performance, keeping the error within $10^{-3}$ radians. Nevertheless, compared to the three proposed controllers, it still exhibits higher error levels. This difference arises because the internal constraints of the synchronization technique facilitate a rapid response, thereby mitigating the effects of the fault. When focusing on the 5th second, it is evident that the peaking phenomenon is effectively reduced in the two proposed controllers utilizing the PFTC scheme, in contrast to AFTC S-TSMC, which displays a slower response to faults due to delayed feedback from the fault estimation. Beyond this point, AFTC S-TSMC and PFTC NN-S-TSMC demonstrate similar performance due to the effectiveness of fault estimation compensation and the online adaptability of the neural network.

Overall, the four controllers exhibit fault tolerance. However, PFTC NN-S-TSMC outperforms the other comparative controllers.

**Figure 4:**
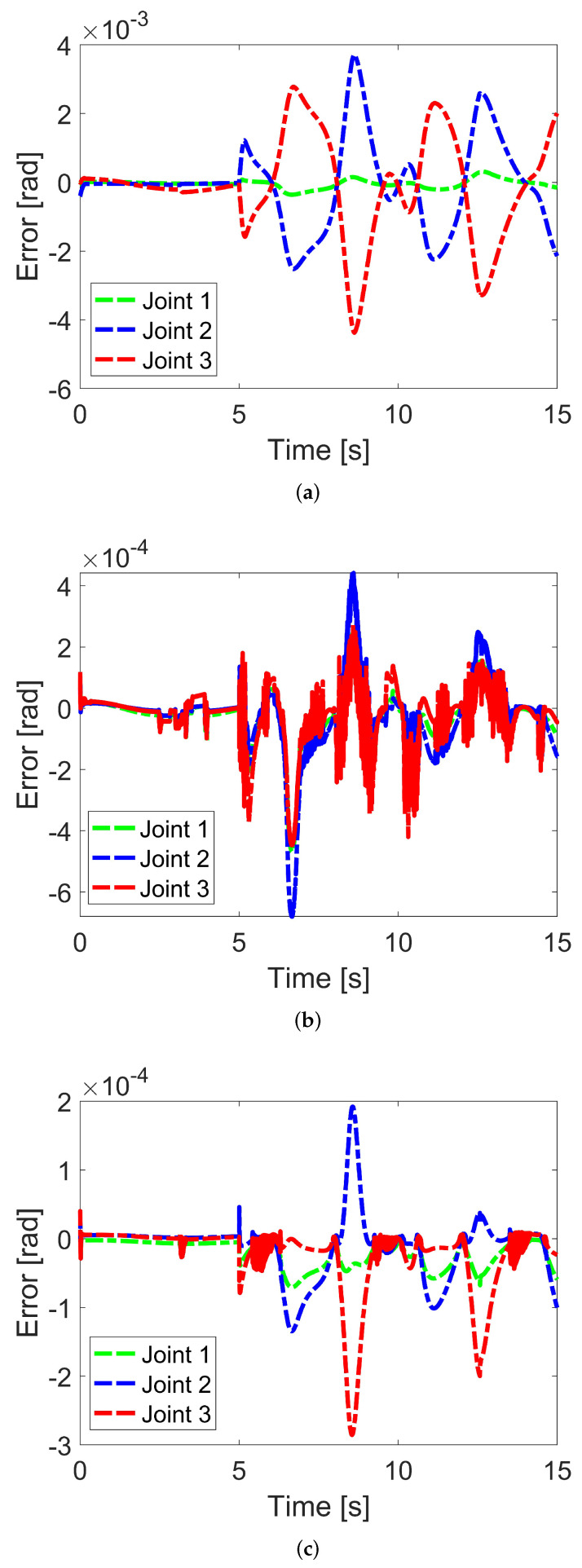
Synchronization performance. (**a**) Conventional TSMC, (**b**) PFTC S-TSMC, (**c**) PFTC NN-S-TSMC.

In Figure 4, the synchronization performance is shown. In Figure 4a, the conventional TSMC does not exhibit synchronization. In Figure 4b,c, synchronization occurs. This shows that the errors at each joint tend to become similar. However, in PFTC NN-S-TSMC, the error trends at each joint occasionally differ due to the online adaptive nature of the neural network. Nevertheless, the effectiveness of the synchronization technique in the two proposed controllers is verified.

**Figure 5:**
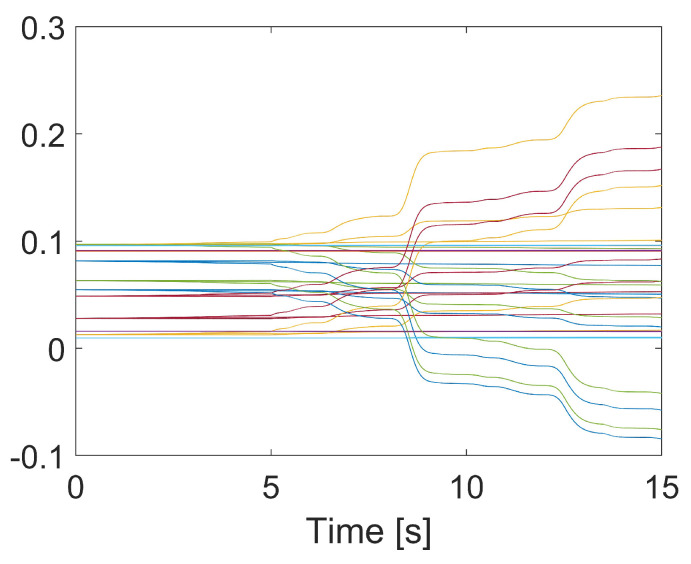
The online update weights of the neural network.

In Figure 5, the weight values of the neural network are presented. It can be seen that before the fault occurs at the 5^th^ second, the weights nearly converged to a constant, as the synchronization errors were relatively small. However, when the fault occurred, the weights were automatically adjusted to suppress the increasing error in the system.

## 5. Discussion and Future Work

Despite the superior fault-tolerant performance of the two proposed controllers, there are certain limitations in their designs. First, the chattering issue in sliding mode control was mitigated using a saturation function, which unfortunately increased the system’s convergence time and introduced a small steady-state error. Second, selecting the control gains and coefficients for synchronization error remains a significant challenge. In this paper, the trial-and-error method used for optimizing the controller parameters is time-consuming and requires both design expertise and in-depth knowledge of the robotic system. These limitations will be addressed in future work.

To reduce chattering, higher-order sliding mode techniques, which are effective at minimizing chattering, accelerating convergence, and eliminating steady-state error, could be investigated. For the parameter selection, methods such as adaptive tuning may be employed to reduce manual effort, while intelligent techniques like neural networks or fuzzy logic could be used to automatically adjust control parameters. Furthermore, it is crucial to explore how synchronization error design parameters impact fault-tolerant control performance.

Applying the synchronization control techniques to real-world robotic systems presents several challenges, such as sensor and actuator noise, computational time, and the practical limitations of sliding mode control. These challenges must be overcome to verify the practical viability of the proposed technique. Finally, expanding the application of this control strategy to multi-robot manipulator systems or multi-quadrotor systems in coordinated tasks will further demonstrate its broader applicability.

## 6. Conclusions

In this paper, two passive fault-tolerant controllers for a robot manipulator are proposed. The first controller, utilizing the robust synchronous terminal sliding mode control, demonstrates an advantage in reducing the peaking phenomenon when compared to active fault-tolerant control architectures. It also performs better than the conventional terminal sliding mode control. This improvement is due to the internal constraints on the joint errors imposed by the synchronization technique, which ensures that the position errors at each joint converge to zero simultaneously. This feature allows the robotic system to quickly and effectively mitigate the impact of faults, maintaining performance even when faults occur. The second proposed controller, which combines the synchronization technique with online adjustments using an orthogonal neural network, further improves fault tolerance by reducing the effects of faults more effectively than both the first proposed controller and the conventional terminal sliding mode control.

## Figures and Tables

**Table 1:** Parameters of 3-DOF Staubli TX60L robot manipulator in MATLAB simulation.

Link	Length (m)	Weight (kg)	Center of Mass (m)	Inertia (${\mathrm{kg}.m}^{2}$)
Link 1	0.217	19.6	[0.05 0 0.09]	Ix = [0.86 0 0.52] Iy = [0 1 0] Iz = [−0.52 0 0.86]
Link 2	0.4	15.2	[−0.01 0 0.1]	Ix = [−0.01 0 1] Iy = [0 −1 0] Iz = [1 0 1]
Link 3	0.45	23.1	[0.05 0 0.08]	Ix = [1 0 0.08] Iy = [−0.01 1 0.08] Iz = [−0.08 −0.08 0.99]

**Table 2:** Mean squared error $\mathrm{MSE}=\frac{1}{n}\sum_{i=1}^{n}{\left(e_{i}\right)}^{2}$ (*n* is the number of samples).

Joints	Conventional TSMC	AFTC S-TSMC [18]	PFTC S-TSMC	PFTC NN-S-TSMC
Joint 1	$1.7027\times 10^{-8}$	$6.0705\times 10^{-11}$	$8.4325\times 10^{-9}$	$8.4292\times 10^{-10}$
Joint 2	$1.5918\times 10^{-6}$	$3.9397\times 10^{-9}$	$1.7215\times 10^{-8}$	$2.2357\times 10^{-9}$
Joint 3	$2.1176\times 10^{-6}$	$5.9448\times 10^{-9}$	$1.0103\times 10^{-8}$	$3.4783\times 10^{-9}$

## Data Availability

Data are contained within the article.

## References

[1] Visinsky M.L., Cavallaro J.R., Walker I.D. (1994). Robotic fault detection and fault tolerance: A survey. Reliab. Eng. Syst. Saf..

[2] Aldridge H.A., Juang J.N. (1997). Experimental Robot Position Sensor Fault Tolerance Using Accelerometers and Joint Torque Sensors.

[3] Le Q.D., Kang H.J. (2020). Implementation of fault-tolerant control for a robot manipulator based on synchronous sliding mode control. Appl. Sci..

[4] Sadeghzadeh I., Mehta A., Chamseddine A., Zhang Y. Active fault tolerant control of a quadrotor uav based on gainscheduled pid control. Proceedings of the 2012 25th IEEE Canadian conference on electrical and computer engineering (CCECE).

[5] Shen Q., Yue C., Goh C.H., Wang D. (2018). Active fault-tolerant control system design for spacecraft attitude maneuvers with actuator saturation and faults. IEEE Trans. Ind. Electron..

[6] Wang R., Wang J. (2012). Passive actuator fault-tolerant control for a class of overactuated nonlinear systems and applications to electric vehicles. IEEE Trans. Veh. Technol..

[7] Benosman M., Lum K.Y. (2009). Passive actuators’ fault-tolerant control for affine nonlinear systems. IEEE Trans. Control. Syst. Technol..

[8] Zabihi M., Mehrizi R.V., Kasaiezadeh A., Pirani M., Khajepour A. (2024). A Hybrid Model-Data Vehicle Sensor and Actuator Fault Detection and Diagnosis System. IEEE Trans. Intell. Transp. Syst..

[9] Berghout T., Benbouzid M. (2024). Fault Diagnosis in Drones via Multiverse Augmented Extreme Recurrent Expansion of Acoustic Emissions with Uncertainty Bayesian Optimisation. Machines.

[10] Zhang Y., Zang Z., Zhang X., Song L., Yu Z., Wang Y., Gao Y., Wang L. (2024). Fault Diagnosis of Industrial Robot Based on Multi-Source Data Fusion and Channel Attention Convolutional Neural Networks. IEEE Access.

[11] Qiu J., Wang T., Sun K., Rudas I.J., Gao H. (2022). Disturbance Observer-Based Adaptive Fuzzy Control for Strict-Feedback Nonlinear Systems With Finite-Time Prescribed Performance. IEEE Trans. Fuzzy Syst..

[12] Xi R.D., Xiao X., Ma T.N., Yang Z.X. (2022). Adaptive Sliding Mode Disturbance Observer Based Robust Control for Robot Manipulators Towards Assembly Assistance. IEEE Robot. Autom. Lett..

[13] Sariyildiz E., Sekiguchi H., Nozaki T., Ugurlu B., Ohnishi K. (2018). A Stability Analysis for the Acceleration-Based Robust Position Control of Robot Manipulators via Disturbance Observer. IEEE/ASME Trans. Mechatronics.

[14] Le Q.D., Kang H.J. (2019). Real Implementation of an Active Fault Tolerant Control Based on Super Twisting Technique for a Robot Manipulator. Proceedings of the Intelligent Computing Methodologies: 15th International Conference, ICIC 2019.

[15] Estrada M.A., Fridman L., Moreno J.A. (2024). Passive fault-tolerant control via sliding-mode-based Lyapunov redesign. IEEE Trans. Autom. Control.

[16] Tan J., Fan Y., Yan P., Wang C., Feng H. (2019). Sliding Mode Fault Tolerant Control for Unmanned Aerial Vehicle with Sensor and Actuator Faults. Sensors.

[17] Alwi H., Edwards C., Edwards C., Lombaerts T., Smaili H. (2010). Fault Tolerant Control Using Sliding Modes with On-Line Control Allocation. Fault Tolerant Flight Control: A Benchmark Challenge.

[18] Le Q.D., Kang H.J. (2020). Finite-Time Fault-Tolerant Control for a Robot Manipulator Based on Synchronous Terminal Sliding Mode Control. Appl. Sci..

[19] Vo A.T., Truong T.N., Le Q.D., Kang H.J. (2023). Fixed-Time Sliding Mode-Based Active Disturbance Rejection Tracking Control Method for Robot Manipulators. Machines.

[20] Jia F., Huang J., He X. (2024). Predefined-Time Fault-Tolerant Control for a Class of Nonlinear Systems With Actuator Faults and Unknown Mismatched Disturbances. IEEE Trans. Autom. Sci. Eng..

[21] Zhou Y., Liu H., Guo H. (2024). L1 Adaptive Fault-Tolerant Control for Nonlinear Systems Subject to Input Constraint and Multiple Faults. Actuators.

[22] Fan H., Fang X., Wang W., Huang J., Liu L. (2023). Adaptive fault-tolerant control for uncertain nonlinear systems with both parameter estimator and controller triggering. Automatica.

[23] Le Q.D., Kang H.J. (2022). An Active Fault-Tolerant Control Based on Synchronous Fast Terminal Sliding Mode for a Robot Manipulator. Actuators.

[24] Koren Y. (1980). Cross-coupled biaxial computer control for manufacturing systems. J. Dyn. Sys. Meas. Control.

[25] Feng L., Koren Y., Borenstein J. (1993). Cross-coupling motion controller for mobile robots. IEEE Control Syst. Mag..

[26] Li C., Yao B., Wang Q. (2018). Modeling and synchronization control of a dual drive industrial gantry stage. IEEE/ASME Trans. Mechatronics.

[27] Ren L., Mills J.K., Sun D. (2007). Experimental comparison of control approaches on trajectory tracking control of a 3-DOF parallel robot. IEEE Trans. Control Syst. Technol..

[28] Le Q.D., Kang H.J., Le T.D., Huang D.S., Hussain A., Han K., Gromiha M.M. (2017). An Adaptive Position Synchronization Controller Using Orthogonal Neural Network for 3-DOF Planar Parallel Manipulators. Intelligent Computing Methodologies.

[29] Shang W., Zhang B., Zhang B., Zhang F., Cong S. (2018). Synchronization control in the cable space for cable-driven parallel robots. IEEE Trans. Ind. Electron..

[30] Cui R., Yan W. (2012). Mutual synchronization of multiple robot manipulators with unknown dynamics. J. Intell. Robot. Syst..

[31] Van M., Sun Y., Mcllvanna S., Nguyen M.N., Khyam M.O., Ceglarek D. (2023). Adaptive Fuzzy Fault Tolerant Control for Robot Manipulators With Fixed-Time Convergence. IEEE Trans. Fuzzy Syst..

[32] Zhang J., Li S., Xiang Z. (2020). Adaptive fuzzy finite-time fault-tolerant control for switched nonlinear large-scale systems with actuator and sensor faults. J. Frankl. Inst..

[33] Ma L., Wang Z., Wang C. (2022). Adaptive neural network state constrained fault-tolerant control for a class of pure-feedback systems with actuator faults. Neurocomputing.

[34] Zheng X., Shen Q. (2020). Neural Network-Based Adaptive Fault-Tolerant Control for a Class of High-Order Strict-Feedback Nonlinear Systems. IEEE Access.

[35] Jin X., Lü S., Yu J. (2022). Adaptive NN-Based Consensus for a Class of Nonlinear Multiagent Systems With Actuator Faults and Faulty Networks. IEEE Trans. Neural Netw. Learn. Syst..

[36] Yam J.Y., Chow T.W. (2000). A weight initialization method for improving training speed in feedforward neural network. Neurocomputing.

[37] Finnoff W., Hergert F., Zimmermann H.G. (1993). Improving model selection by nonconvergent methods. Neural Netw..

[38] Qian N. (1999). On the momentum term in gradient descent learning algorithms. Neural Netw..

[39] Kayacan E., Khanesar M.A., Kayacan E., Khanesar M.A. (2016). Chapter 5—Gradient Descent Methods for Type-2 Fuzzy Neural Networks. Fuzzy Neural Networks for Real Time Control Applications.

[40] Yang S.S., Tseng C.S. (1996). An orthogonal neural network for function approximation. IEEE Trans. Syst. Man Cybern. Part B Cybern..

[41] Li S., Jia K., Wen Y., Liu T., Tao D. (2021). Orthogonal Deep Neural Networks. IEEE Trans. Pattern Anal. Mach. Intell..

[42] Wang J., Chen Y., Chakraborty R., Yu S.X. Orthogonal Convolutional Neural Networks. Proceedings of the IEEE/CVF Conference on Computer Vision and Pattern Recognition (CVPR).

[43] Mashhadi P.S., Nowaczyk S., Pashami S. (2021). Parallel orthogonal deep neural network. Neural Netw..

[44] Perić S.L., Antić D.S., Milovanović M.B., Mitić D.B., Milojković M.T., Nikolić S.S. (2016). Quasi-Sliding Mode Control With Orthogonal Endocrine Neural Network-Based Estimator Applied in Anti-Lock Braking System. IEEE/ASME Trans. Mechatronics.

[45] Le Q.D., Kang H.J., Le T.D. (2016). An Adaptive Controller with An Orthogonal Neural Network and A Third Order Sliding Mode Observer for Robot Manipulators. Int. J. Mech. Eng. Robot. Res..

[46] Yu X., Zhihong M. (2002). Fast terminal sliding-mode control design for nonlinear dynamical systems. IEEE Trans. Circuits Syst. Fundam. Theory Appl..

[47] Liu H., Zhang T. (2013). Neural network-based robust finite-time control for robotic manipulators considering actuator dynamics. Robot. Comput.-Integr. Manuf..

[48] Rodd M.G., Craig J.J. (1987). Introduction to Robotics: Mechanics and Control.

[49] Bošković J.D., Mehra R.K. (2003). Failure detection, identification and reconfiguration system for a redundant actuator assembly. IFAC Proc. Vol..

[50] Courant R., Hilbert D.R. (1947). Methods of Mathematical Physics. Math. Gaz..

[51] Chen X., Zhang Q., Sun Y. (2019). Non-kinematic calibration of industrial robots using a rigid–flexible coupling error model and a full pose measurement method. Robot. Comput.-Integr. Manuf..

